# Convergence and stability of the exponential Euler method for semi-linear stochastic delay differential equations

**DOI:** 10.1186/s13660-017-1518-5

**Published:** 2017-10-06

**Authors:** Ling Zhang

**Affiliations:** Mathematical Department of Teacher Education Institute, DaQing Normal University, DaQing, 163712 P.R. China

**Keywords:** 65F20, stochastic delay differential equation, exponential Euler method, Lipschitz condition, Itô formula, strong convergence

## Abstract

The main purpose of this paper is to investigate the strong convergence and exponential stability in mean square of the exponential Euler method to semi-linear stochastic delay differential equations (SLSDDEs). It is proved that the exponential Euler approximation solution converges to the analytic solution with the strong order $\frac{1}{2}$ to SLSDDEs. On the one hand, the classical stability theorem to SLSDDEs is given by the Lyapunov functions. However, in this paper we study the exponential stability in mean square of the exact solution to SLSDDEs by using the definition of logarithmic norm. On the other hand, the implicit Euler scheme to SLSDDEs is known to be exponentially stable in mean square for any step size. However, in this article we propose an explicit method to show that the exponential Euler method to SLSDDEs is proved to share the same stability for any step size by the property of logarithmic norm.

## Introduction

Stochastic modeling has come to play an important role in many branches of science and industry. Such models have been used with great success in a variety of application areas, including biology, epidemiology, mechanics, economics and finance. Most stochastic differential equations (SDEs) are nonlinear and cannot be solved explicitly, whence numerical solutions are required in practice. Numerical solutions to SDEs have been discussed under the Lipschitz condition and the linear growth condition by many authors (see [1–7]). Higham et al. [2] gave almost sure and moment exponential stability in the numerical simulation of SDEs. Many authors have discussed numerical solutions to stochastic delay differential equations (SDDES) (see [8–12]). Cao et al. [8] obtained MS-stability of the Euler-Maruyama method for SDDEs. Mao [12] discussed exponential stability of equidistant Euler-Maruyama approximations of SDDES. The explicit Euler scheme is most commonly used for approximating SDEs with the global Lipschitz condition. Unfortunately, the step size of Euler method for SDEs has limits for research of stability. Therefore, the stability of the implicit Euler scheme to SDEs is known for any step size. However, in this article we propose an explicit method to show that the exponential Euler method to SLSDDEs is proved to share the stability for any step size by the property of logarithmic norm.

The paper is organized as follows. In Section 2, we introduce necessary notations and the exponential Euler method. In Section 3, we obtain the convergence of the exponential Euler method to SLSDDEs under Lipschitz condition and the linear growth condition. In Section 4, we obtain the exponential stability in mean square of the exponential Euler method to SLSDDEs. Finally, two examples are provided to illustrate our theory.

## Preliminary notation and the exponential Euler method

In this paper, unless otherwise specified, let $\vert x \vert $ be the Euclidean norm in $x\in R^{n}$. If *A* is a vector or matrix, its transpose is denoted by $A^{T}$. If *A* is a matrix, its trace norm is denoted by $\vert A \vert =\sqrt{\operatorname{trace}(A^{T}A)}$. For simplicity, we also denote $a\wedge b=\min\{a,b\}$, $a\vee b=\max\{a,b\}$.

Let $(\Omega,\mathbf{F},P)$ be a complete probability space with a filtration $\{\mathbf{F}_{t}\}_{t\geq0}$, satisfying the usual conditions. $\mathbf{L}^{1}([0,\infty),R^{n})$ and $\mathbf{L}^{2}([0,\infty),R^{n})$ denote the family of all real-valued $\mathbf{F}_{t}$-adapted processes $f(t)_{t\geq0}$ such that for every $T>0$, $\int_{0}^{T} \vert f(t) \vert \,dt<\infty$ a.s. and $\int_{0}^{T} \vert f(t) \vert ^{2}\,dt<\infty$ a.s., respectively. For any $a,b\in R$ with $a< b$, denote by $C([a,b];R^{n})$ the family of continuous functions *ϕ* from $[a,b]$ to $R^{n}$ with the norm $\Vert \phi \Vert =\sup_{a\leq\theta\leq b} \vert \phi (\theta) \vert $. Denote by $C_{\mathbf{F}_{t}}^{b}([a,b];R^{n})$ the family of all bounded $\mathbf{F}_{t}$-measurable $C([a,b];R^{n})$-valued random variables. Let $B(t)=(B_{1}(t),\ldots,B_{d}(t))^{T}$ be a *d*-dimensional Brownian motion defined on the probability space $(\Omega,\mathbf{F},P)$. Throughout this paper, we consider the following semi-linear stochastic delay differential equations:

2.1 $$ \textstyle\begin{cases} dx(t)=(Ax(t)+f(t,x(t),x(t-\tau)))\,dt+g(t,x(t),x(t-\tau))\,d B(t),& t\in[0,T],\\ x(t)=\xi(t),&t\in[-\tau,0], \end{cases} $$

where $T>0$, $\tau>0$, $\{\xi(t), t\in[-\tau,0]\}=\xi\in C^{b}_{\mathbf {F}_{0}}([-\tau,0];R^{n})$, $f: R^{+}\times R^{n}\times R^{n} \rightarrow R^{n}$, $g: R^{+}\times R^{n}\times R^{n}\rightarrow R^{n\times d}$, $A\in R^{n\times n}$ is the matrix [13]. By the definition of stochastic differential, this equation is equivalent to the following stochastic integral equation:

2.2 $$\begin{aligned} x(t) =&e^{At}\xi+ \int_{0}^{t}e^{A(t-s)}f\bigl(s,x(s),x(s-\tau) \bigr)\,ds \\ &{}+ \int_{0}^{t}e^{A(t-s)}g\bigl(s,x(s),x(s-\tau) \bigr)\,d B(s) \quad\forall t\geq0. \end{aligned}$$

Moreover, we also require the coefficients *f* and *g* to be sufficiently smooth.

To be precise, let us state the following conditions.

(H1): (The Lipschitz condition) There exists a positive constant $L_{1}$ such that
  $$\bigl\vert f(t,x,y)-f(t,\bar{x},\bar{y}) \bigr\vert ^{2}\vee\vert g(t,x,y)-g(t,\bar{x}, \bar{y}) \bigl\vert ^{2}\leq L_{1} \bigl( \bigr\vert x-\bar{x} \bigl\vert ^{2}+ \bigr\vert y-\bar{y} \vert^{2}\bigr) $$
  for those $x, \bar{x}, y, \bar{y}\in R^{n}$.
(H2): (Linear growth condition) There exists a positive constant $L_{2}$ such that
  $$\bigl\vert f(t,x,y) \bigr\vert ^{2}\vee \bigl\vert g(t,x,y) \bigr\vert ^{2}\leq L_{2}\bigl(1+ \vert x \vert ^{2}+ \vert y \vert ^{2}\bigr) $$
  for all $(t,x,y)\in R^{+}\times R^{n}\times R^{n}$.
(H3): *f* and *g* are supposed to satisfy the following property:
  $$\bigl\vert f(s,x,y)-f(t,x,y) \bigr\vert ^{2}\vee \bigl\vert g(s,x,y)-g(t,x,y) \bigr\vert ^{2}\leq K_{1}\bigl(1+ \vert x \vert ^{2}+ \vert y \vert ^{2}\bigr) \vert s-t \vert , $$
  where $K_{1}$ is a constant and $s,t\in[0,T]$ with $t>s$.

Let $h=\frac{\tau}{m}$ be a given step size with integer $m\geq1$, and let the gridpoints $t_{n}$ be defined by $t_{n}=nh$ ($n=0,1,2,\ldots$). We consider the exponential Euler method to (2.1)

2.3 $$\begin{aligned} y_{n+1}=e^{Ah}y_{n}+e^{Ah}f(t_{n},y_{n},y_{n-m})h+e^{Ah}g(t_{n},y_{n},y_{n-m}) \Delta B_{n}, \end{aligned}$$

where $\Delta B_{n}=B(t_{n})-B(t_{n-1})$, $n=0,1,2,\ldots , y_{n}$, is approximation to the exact solution $x(t_{n})$. The continuous exponential Euler method approximate solution is defined by

2.4 $$\begin{aligned} y(t) =&e^{At}\xi+ \int^{t}_{0}e^{A(t-\underline {s})}f\bigl(\underline{s},z(s),z(s-\tau)\bigr)\,d s \\ &{}+ \int^{t}_{0}e^{A(t-\underline{s})}g\bigl(\underline {s},z(s),z(s-\tau)\bigr)\,dB(s), \end{aligned}$$

where $\underline{s}=[\frac{s}{h}]h$ and $[x]$ denotes the largest integer which is smaller than *x*, $z(t)=\sum^{\infty}_{k=0}y_{k}1_{[kh,(k+1)h)}(t)$ with $1_{\mathcal{A}}$ denoting the indicator function for the set $\mathcal {A}$. It is not difficult to see that $y(t_{n})=z(t_{n})=y_{n}$ for $n=0,1,2,\ldots$ . That is, the step function $z(t)$ and the continuous exponential Euler solution $y(t)$ coincide with the discrete solution at the gridpoint. Let $C^{2,1}(R^{n}\times R_{+};R)$ denote the family of all continuous nonnegative functions $V(x,t)$ defined on $R^{n}\times R_{+}$ such that they are continuously twice differentiable in *x* and once in *t*. Given $V\in C^{2,1}(R^{n}\times R_{+};R)$, we define the operator $\mathcal {L}V:R^{n}\times R^{n}\times R_{+}\rightarrow R$ by

$$\begin{aligned} \mathcal{L}V(x,y,t)=V_{t}(x,t)+V_{x}(x,t)f(x,y,t)+ \frac {1}{2}\operatorname{trace}\bigl[g^{T}(x,y,t)V_{xx}(x)g(x,y,t) \bigr], \end{aligned}$$

where

$$\begin{gathered} V_{t}(x,t)=\frac{\partial V(x,t)}{\partial t}, \qquad V_{x}(x,t)=\biggl(\frac {\partial V(x,t)}{\partial x_{1}},\ldots,\frac{\partial V(x,t)}{\partial x_{n}}\biggr), \\ V_{xx}(x,t)=\biggl(\frac{\partial^{2} V(x,t)}{\partial x_{i} \,\partial x_{j}}\biggr)_{n\times n}. \end{gathered} $$

Let us emphasize that $\mathcal{L}V$ is defined on $R^{n}\times R^{n}\times R_{+}$, while *V* is only defined on $R^{n}\times R_{+}$.

## Convergence of the exponential Euler method

We will show the strong convergence of the exponential Euler method (2.4) on equations (2.1).

### Theorem 3.1

*Under conditions* (H1), (H2) *and* (H3), *the exponential Euler method approximate solution converges to the exact solution of equations* (2.1) *in the sense that*

3.1 $$\begin{aligned} \lim_{h\rightarrow0}E\Bigl[\sup_{0\leq t\leq T} \bigl\vert y(t)-x(t) \bigr\vert ^{2}\Bigr]=0. \end{aligned}$$

In order to prove this theorem, we first prepare two lemmas.

### Lemma 3.1

*Under the linear growth condition* (H2), *there exists a positive constant* $C_{1}$ *such that the solution of equations* (2.1) *and the continuous exponential Euler method approximate solution* (2.4) *satisfy*

3.2 $$\begin{aligned} E\sup_{-\tau\leq t\leq T} \bigl\vert y(t) \bigr\vert ^{2}\vee E\sup_{-\tau\leq t\leq T} \bigl\vert x(t) \bigr\vert ^{2}\leq C_{1}\bigl(1+E \vert \xi \vert ^{2}\bigr), \end{aligned}$$

*where* $C_{1}=\max\{3e^{2 \vert A \vert T}e^{6e^{2 \vert A \vert T}T(T+4)L_{2}},e^{2 \vert A \vert T}T(T+4)L_{2}e^{6e^{2 \vert A \vert T}T(T+4)L_{2}}\}$ *is a constant independent of* *h*.

### Proof

It follows from (2.4) that

3.3 $$\begin{aligned} \bigl\vert y(t) \bigr\vert ^{2} =&\biggl\vert e^{At}\xi+ \int^{t}_{0}e^{A(t-\underline{s})}f\bigl(\underline {s},z(s),z(s-\tau)\bigr)\,d s \\ &{}+ \int^{t}_{0}e^{A(t-\underline{s})}g\bigl(\underline{s},z(s),z(s-\tau )\bigr)\,dB(s)\biggr\vert ^{2} \\ \leq&3\biggl[ \bigl\vert e^{At}\xi \bigr\vert ^{2}+ \biggl\vert \int ^{t}_{0}e^{A(t-\underline{s})}f\bigl(\underline{s},z(s),z(s-\tau)\bigr)\,d s \biggr\vert ^{2} \\ &{}+ \biggl\vert \int^{t}_{0}e^{A(t-\underline{s})}g\bigl(\underline {s},z(s),z(s-\tau)\bigr)\,dB(s) \biggr\vert ^{2}\biggr]. \end{aligned}$$

By Hölder’s inequality, we obtain

3.4 $$\begin{aligned} \bigl\vert y(t) \bigr\vert ^{2} \leq&3\biggl[ \bigl\vert e^{At}\xi \bigr\vert ^{2}+T \int_{0}^{t} \bigl\vert e^{A(t-\underline {s})}f\bigl(\underline{s},z(s),z(s-\tau)\bigr) \bigr\vert ^{2}\,ds \\ &{}+ \biggl\vert \int_{0}^{t}e^{A(t-\underline{s})}g\bigl(\underline {s},z(s),z(s-\tau)\bigr)\,dB(s) \biggr\vert ^{2}\biggr]. \end{aligned}$$

This implies that for any $0\leq t_{1}\leq T$,

3.5 $$\begin{aligned}& E\sup_{0\leq t\leq t_{1}} \bigl\vert y(t) \bigr\vert ^{2} \\& \quad \leq 3\biggl[E\sup_{0\leq t\leq t_{1}} \bigl\vert e^{At}\xi \bigr\vert ^{2}+TE\sup_{0\leq t\leq t_{1}} \int_{0}^{t} \bigl\vert e^{A(t-\underline{s})}f\bigl(\underline {s},z_{1}(s),z_{2}(s)\bigr) \bigr\vert ^{2}\,ds \\& \qquad {}+E\sup_{0\leq t\leq t_{1}} \biggl\vert \int_{0}^{t}e^{A(t-\underline{s})}g\bigl(\underline {s},z(s),z(s-\tau)\bigr)\,dB(s) \biggr\vert ^{2}\biggr] \\& \quad \leq 3\biggl[E\sup_{0\leq t\leq t_{1}} \bigl\vert e^{At} \bigr\vert ^{2} \vert \xi \vert ^{2}+TE\sup _{0\leq t\leq t_{1}} \int_{0}^{t} \bigl\vert e^{A(t-\underline{s})} \bigr\vert ^{2} \bigl\vert f\bigl(\underline{s},z(s),z(s-\tau)\bigr) \bigr\vert ^{2}\,ds \\& \qquad {}+E\sup_{0\leq t\leq t_{1}} \bigl\vert e^{At} \bigr\vert ^{2} \biggl\vert \int_{0}^{t}e^{-A\underline {s}}g\bigl(\underline{s},z(s),z(s-\tau)\bigr)\,dB(s) \biggr\vert ^{2}\biggr]. \end{aligned}$$

By Doob’s martingale inequality, it is not difficult to show that

3.6 $$ \begin{aligned}[b] E\sup_{0\leq t\leq t_{1}} \bigl\vert y(t) \bigr\vert ^{2} & \leq 3\biggl[e^{2 \vert A \vert T}E \vert \xi \vert ^{2} \\ &\quad {}+Te^{2 \vert A \vert T}E \int_{0}^{t_{1}} \bigl\vert f\bigl(\underline{s},z(s),z(s-\tau)\bigr) \bigr\vert ^{2}\,ds \\ &\quad {}+4e^{2 \vert A \vert T}E \int_{0}^{t_{1}} \bigl\vert e^{-A\underline{s}}g\bigl(\underline{s},z(s),z(s-\tau)\bigr) \bigr\vert ^{2}\,ds\biggr] \\ &\leq3e^{2 \vert A \vert T}\biggl[E \vert \xi \vert ^{2}+TE \int_{0}^{t_{1}} \bigl\vert f\bigl(\underline{s},z(s),z(s-\tau)\bigr) \bigr\vert ^{2}\,ds \\ &\quad {}+4e^{2 \vert A \vert T}E \int_{0}^{t_{1}} \bigl\vert g\bigl(\underline{s},z(s),z(s-\tau)\bigr) \bigr\vert ^{2}\,ds\biggr]. \end{aligned} $$

Making use of (H2) yields

3.7 $$\begin{aligned} E\sup_{0\leq t\leq t_{1}} \bigl\vert y(t) \bigr\vert ^{2} \leq&3e^{2 \vert A \vert T}\biggl[E \Vert \xi \Vert ^{2} \\ &{}+\bigl(T+4e^{2 \vert A \vert T}\bigr)L_{2}E \int _{0}^{t_{1}}\bigl(1+ \bigl\vert z(s) \bigr\vert ^{2}+ \bigl\vert z(s-\tau) \bigr\vert ^{2} \bigr)\,ds\biggr] \\ \leq&3e^{2 \vert A \vert T}E \Vert \xi \Vert ^{2}+3e^{2 \vert A \vert T}T \bigl(T+4e^{2 \vert A \vert T}\bigr)L_{2} \\ &{}+6e^{2 \vert A \vert T}\bigl(T+4e^{2 \vert A \vert T}\bigr)L_{2} \int_{0}^{t_{1}}E\sup_{-\tau\leq u\leq s} \bigl\vert y(u) \bigr\vert ^{2}\,ds. \end{aligned}$$

Thus

3.8 $$\begin{aligned} E\sup_{-\tau\leq t\leq t_{1}} \bigl\vert y(t) \bigr\vert ^{2} \leq&3e^{2 \vert A \vert T}E \Vert \xi \Vert ^{2}+3e^{2 \vert A \vert T}T \bigl(T+4e^{2 \vert A \vert T}\bigr)L_{2} \\ &{}+6e^{2 \vert A \vert T}\bigl(T+4e^{2 \vert A \vert T}\bigr)L_{2} \int_{0}^{t_{1}}E\sup_{-\tau\leq u\leq s} \bigl\vert y(u) \bigr\vert ^{2}\,ds. \end{aligned}$$

By Gronwall’s inequality, we get

3.9 $$\begin{aligned} E\sup_{-\tau\leq t\leq T} \bigl\vert y(t) \bigr\vert ^{2} \leq C_{1}, \end{aligned}$$

where $C_{1}=(3e^{2 \vert A \vert T}E \Vert \xi \Vert ^{2}+3e^{2 \vert A \vert T}T(T+4e^{2 \vert A \vert T})L_{2})e^{6e^{2 \vert A \vert T}T(T+4e^{2 \vert A \vert T})L_{2}}$. In the same way, we obtain

3.10 $$\begin{aligned} E\sup_{-\tau\leq t\leq T} \bigl\vert x(t) \bigr\vert ^{2} \leq C_{1}, \end{aligned}$$

where $C_{1}=(3e^{2 \vert A \vert T}E \Vert \xi \Vert ^{2}+3e^{2 \vert A \vert T}T(T+4e^{2 \vert A \vert T})L_{2})e^{6e^{2 \vert A \vert T}T(T+4e^{2 \vert A \vert T})L_{2}}$. The proof is completed. □

The following lemma shows that both $y(t)$ and $z(t)$ are close to each other.

### Lemma 3.2

*Under condition* (H2). *Then*

3.11 $$\begin{aligned} E \bigl\vert y(t)-z(t) \bigr\vert ^{2}\leq C_{2}(\xi)h, \quad\forall t\in[0,T], \end{aligned}$$

*where* $C_{2}(\xi)$ *is a constant independent of* *h*.

### Proof

For $t\in[0,T]$, there is an integer *k* such that $t\in[t_{k},t_{k+1})$. We compute

3.12 $$\begin{aligned} \bigl\vert y(t)-z(t) \bigr\vert ^{2} \leq&3\bigl[ \bigl\vert e^{A(t-t_{k})}-I \bigr\vert ^{2} \vert y_{k} \vert ^{2}+ \bigl\vert e^{A(t-t_{k})}f(t_{k},y_{k},y_{k-m}) (t-t_{k}) \bigr\vert ^{2} \\ &{}+ \bigl\vert e^{A(t-t_{k})}g(t_{k},y_{k},y_{k-m}) \bigl(B(t)-B(t_{k})\bigr) \bigr\vert ^{2}\bigr] \\ \leq&3\bigl[ \bigl\vert e^{A(t-t_{k})}-I \bigr\vert ^{2} \vert y_{k} \vert ^{2}+ \bigl\vert e^{A(t-t_{k})} \bigr\vert ^{2} \bigl\vert f(t_{k},y_{k},y_{k-m}) \bigr\vert ^{2} \bigl\vert (t-t_{k}) \bigr\vert ^{2} \\ &{}+ \bigl\vert e^{A(t-t_{k})} \bigr\vert ^{2} \bigl\vert g(t_{k},y_{k},y_{k-m}) \bigr\vert ^{2} \bigl\vert \bigl(B(t)-B(t_{k})\bigr) \bigr\vert ^{2}\bigr], \end{aligned}$$

where *I* is an identity matrix. Taking the expectation of both sides, we can see

3.13 $$\begin{aligned} E \bigl\vert y(t)-z(t) \bigr\vert ^{2} \leq&3\bigl[ \bigl\vert e^{A(t-t_{k})}-I \bigr\vert ^{2}E \vert y_{k} \vert ^{2}+h^{2}e^{2 \vert A \vert T}E \bigl\vert f(t_{k},y_{k},y_{k-m}) \bigr\vert ^{2} \\ &{}+he^{2 \vert A \vert T}E \bigl\vert g(t_{k},y_{k},y_{k-m}) \bigr\vert ^{2}\bigr]. \end{aligned}$$

Using the linear growth conditions, we have

3.14 $$\begin{aligned} E \bigl\vert y(t)-z(t) \bigr\vert ^{2} \leq&3\bigl[ \bigl\vert e^{A(t-t_{k})}-I \bigr\vert ^{2}E \vert y_{k} \vert ^{2}+h^{2}e^{2 \vert A \vert T}L_{2}E\bigl(1+ \vert y_{k} \vert ^{2}+ \vert y_{k-m} \vert ^{2}\bigr) \\ &{}+he^{2 \vert A \vert T}L_{2}E\bigl(1+ \vert y_{k} \vert ^{2}+ \vert y_{k-m} \vert ^{2}\bigr)\bigr] \\ \leq&3\bigl[ \bigl\vert e^{A(t-t_{k})}-I \bigr\vert ^{2}C_{1}+ \bigl(h^{2}+h\bigr)e^{2 \vert A \vert T}L_{2}(1+2C_{1}) \bigr]. \end{aligned}$$

Since $\vert e^{A(t-t_{k})}-I_{k} \vert \leq e^{ \vert A \vert h}-1\leq \vert A \vert he^{ \vert A \vert h}\leq \vert A \vert he^{ \vert A \vert T}$, we have

3.15 $$\begin{aligned} E \bigl\vert y(t)-z(t) \bigr\vert ^{2}\leq C_{2}(\xi)h, \end{aligned}$$

where $C_{2}(\xi)=3 \vert A \vert ^{2}Te^{2 \vert A \vert T}C_{1}+3(T+1)e^{2 \vert A \vert T}L_{2}(1+2C_{1})$ is a constant independent of *h*. The proof is completed. □

### Proof of Theorem 3.1

By (2.2) and (2.4), we have

3.16 $$\begin{aligned}& \bigl\vert x(t)-y(t) \bigr\vert ^{2} \\& \quad \leq 2 \biggl\vert \int_{0}^{t}\bigl[e^{A(t-s)}f\bigl(s,x(s),x(s- \tau )\bigr)-e^{A(t-\underline{s})}f\bigl(\underline{s},z(s),z(s-\tau)\bigr)\bigr]\,ds \biggr\vert ^{2} \\& \qquad {}+2 \biggl\vert \int_{0}^{t}\bigl[e^{A(t-s)}g\bigl(s,x(s),x(s- \tau)\bigr) \\& \qquad {}-e^{A(t-\underline{s})}g\bigl(\underline{s},z(s),z(s-\tau)\bigr)\bigr]\,d B(s) \biggr\vert ^{2}. \end{aligned}$$

By Hölder’s inequality, we obtain

3.17 $$ \begin{gathered}[b] \bigl\vert x(t)-y(t) \bigr\vert ^{2} \\ \quad \leq 6T \int_{0}^{t} \bigl\vert e^{A(t-s)}f \bigl(s,x(s),x(s-\tau)\bigr)-e^{A(t-\underline {s})}f\bigl(s,x(s),x(s-\tau)\bigr) \bigr\vert ^{2}\,ds \\ \qquad {}+6T \int_{0}^{t} \bigl\vert e^{A(t-\underline {s})}f \bigl(s,x(s),x(s-\tau)\bigr) \\ \qquad {}-e^{A(t-\underline{s})}f\bigl(s,z(s),z(s-\tau)\bigr) \bigr\vert ^{2}\,ds \\ \qquad {}+6T \int_{0}^{t} \bigl\vert e^{A(t-\underline {s})}f \bigl(s,z(s),z(s-\tau)\bigr)-e^{A(t-\underline{s})}f\bigl(\underline {s},z(s),z(s-\tau) \bigr) \bigr\vert ^{2}\,ds \\ \qquad {}+6 \biggl\vert \int_{0}^{t}\bigl[e^{A(t-s)}g\bigl(s,x(s),x(s- \tau )\bigr) \\ \qquad {}-e^{A(t-\underline{s})}g\bigl(s,x(s),x(s-\tau )\bigr)\bigr]\,dB(s) \biggr\vert ^{2} \\ \qquad {}+6 \biggl\vert \int_{0}^{t}\bigl[e^{A(t-\underline {s})}g\bigl(s,x(s),x(s- \tau)\bigr)-e^{A(t-\underline{s})}g\bigl(s,z(s),z(s-\tau )\bigr)\bigr]\,dB(s) \biggr\vert ^{2} \\ \qquad {}+6 \biggl\vert \int_{0}^{t}\bigl[e^{A(t-\underline {s})}g\bigl(s,z(s),z(s- \tau)\bigr) \\ \qquad {}-e^{A(t-\underline {s})}g\bigl(\underline{s},z(s),z(s-\tau)\bigr)\bigr]\,dB(s) \biggr\vert ^{2}. \end{gathered} $$

This implies that for any $0\leq t_{1}\leq T$, by Doob’s martingale inequality, we have

3.18 $$\begin{aligned} E\sup_{0\leq t\leq t_{1}} \bigl\vert x(t)-y(t) \bigr\vert ^{2} \leq&6TE\sup_{0\leq t\leq t_{1}} \int_{0}^{t} \bigl\vert e^{A(t-s)}f \bigl(s,x(s),x(s-\tau)\bigr) \\ &{}-e^{A(t-\underline{s})}f\bigl(s,x(s),x(s-\tau)\bigr) \bigr\vert ^{2}\,ds \\ &{}+6TE\sup_{0\leq t\leq t_{1}} \int_{0}^{t}E \bigl\vert e^{A(t-\underline{s})}f \bigl(s,x(s),x(s-\tau )\bigr) \\ &{}-e^{A(t-\underline{s})}f\bigl(s,z(s),z(s-\tau )\bigr) \bigr\vert ^{2}\,ds \\ &{}+6TE\sup_{0\leq t\leq t_{1}} \int_{0}^{t}E \bigl\vert e^{A(t-\underline{s})}f \bigl(s,z(s),z(s-\tau )\bigr) \\ &{}-e^{A(t-\underline{s})}f\bigl(\underline {s},z(s),z(s-\tau)\bigr) \bigr\vert ^{2}\,ds \\ &{}+6E\sup_{0\leq t\leq t_{1}} \bigl\vert e^{At} \bigr\vert ^{2} \biggl\vert \int _{0}^{t}e^{-As}g\bigl(s,x(s),x(s- \tau)\bigr) \\ &{}-e^{-A\underline{s}}g\bigl(s,x(s),x(s-\tau)\bigr)\,dB(s) \biggr\vert ^{2} \\ &{}+6E\sup_{0\leq t\leq t_{1}} \bigl\vert e^{At} \bigr\vert ^{2} \biggl\vert \int_{0}^{t}e^{-A\underline {s}}g\bigl(s,x(s),x(s-\tau) \bigr) \\ &{}-e^{-A\underline {s}}g\bigl(s,z(s),z(s-\tau)\bigr)\,dB(s) \biggr\vert ^{2} \\ &{}+6E\sup_{0\leq t\leq t_{1}} \bigl\vert e^{At} \bigr\vert ^{2} \biggl\vert \int_{0}^{t}e^{-A\underline {s}}g\bigl(s,z(s),z(s-\tau) \bigr) \\ &{}-e^{-A\underline {s}}g\bigl(\underline{s},z(s),z(s-\tau)\bigr)\,dB(s) \biggr\vert ^{2}. \end{aligned}$$

We compute the first item in (3.18)

3.19 $$\begin{aligned}& E\sup_{0\leq t\leq t_{1}} \int_{0}^{t}E \bigl\vert e^{A(t-s)}f \bigl(s,x(s),x(s-\tau)\bigr) \\& \qquad {}-e^{A(t-\underline{s})}f\bigl(s,x(s),x(s-\tau)\bigr) \bigr\vert ^{2}\,ds \\& \quad \leq E\sup_{0\leq t\leq t_{1}} \int_{0}^{t} \bigl\vert e^{A(t-s)}-e^{A(t-\underline{s})} \bigr\vert ^{2}E \bigl\vert f\bigl(s,x(s),x(s-\tau)\bigr) \bigr\vert ^{2}\,ds \\& \quad \leq L_{2}E\sup_{0\leq t\leq t_{1}} \int_{0}^{t} \bigl\vert e^{A(t-\underline{s})} \bigr\vert ^{2} \bigl\vert e^{A(\underline {s}-s)}-I \bigr\vert ^{2}E\bigl(1+ \bigl\vert x(s) \bigr\vert ^{2}+ \bigl\vert x(s-\tau) \bigr\vert ^{2}\bigr)\,ds \\& \quad \leq L_{2}e^{2 \vert A \vert T}T \bigl\vert e^{A(\underline{s}-s)}-I \bigr\vert ^{2}(1+2C_{1}). \end{aligned}$$

We compute the following two formulas in (3.18):

3.20 $$\begin{aligned}& E\sup_{0\leq t\leq t_{1}} \int_{0}^{t}E \bigl\vert e^{A(t-\underline{s})}f \bigl(s,x(s),x(s-\tau )\bigr)-e^{A(t-\underline{s})}f\bigl(s,z(s),z(s-\tau)\bigr) \bigr\vert ^{2}\,ds \\& \quad \leq L_{1}e^{2 \vert A \vert T} \int_{0}^{t_{1}}E\bigl( \bigl\vert x(s)-z(s) \bigr\vert ^{2}+ \bigl\vert x(s-\tau)-z_{2}(s) \bigr\vert ^{2}\bigr)\,ds \\& \quad \leq 2L_{1}e^{2 \vert A \vert T} \int_{0}^{t_{1}}E\bigl( \bigl\vert x(s)-y(s) \bigr\vert ^{2}+ \bigl\vert y(s)-z(s) \bigr\vert ^{2} \\& \qquad {}+ \bigl\vert x(s-\tau)-y(s-\tau) \bigr\vert ^{2}+ \bigl\vert y(s-\tau)-z(s-\tau) \bigr\vert ^{2}\bigr)\,ds \\& \quad \leq 4L_{1}e^{2 \vert A \vert T}TC_{2}(\xi)h \\& \qquad {}+2L_{1}e^{2 \vert A \vert T} \int_{0}^{t_{1}}E\bigl( \bigl\vert x(s)-y(s) \bigr\vert ^{2}+ \bigl\vert x(s-\tau)-y(s-\tau) \bigr\vert ^{2}\bigr)\,ds \end{aligned}$$

and

3.21 $$\begin{aligned}& E\sup_{0\leq t\leq t_{1}} \int_{0}^{t}E \bigl\vert e^{A(t-\underline{s})}f \bigl(s,z(s),z(s-\tau )\bigr) \\& \qquad {}-e^{A(t-\underline{s})}f\bigl(\underline {s},z(s),z(s-\tau)\bigr) \bigr\vert ^{2}\,ds \\& \quad \leq K_{1} e^{2 \vert A \vert T}TE\bigl(1+ \bigl\vert z(s) \bigr\vert ^{2}+ \bigl\vert z(s-\tau) \bigr\vert ^{2}\bigr)h \\& \quad \leq K_{1} e^{2 \vert A \vert T}T(1+2C_{1})h. \end{aligned}$$

In the same way, we can obtain

3.22 $$\begin{aligned}& E\sup_{0\leq t\leq t_{1}} \bigl\vert e^{At} \bigr\vert ^{2} \biggl\vert \int _{0}^{t}e^{-As}g\bigl(s,x(s),x(s- \tau)\bigr) \\& \qquad {}-e^{-A\underline{s}}g\bigl(s,x(s),x(s-\tau)\bigr)\,dB(s) \biggr\vert ^{2} \\& \quad \leq 4e^{2 \vert A \vert T}E \int_{0}^{t_{1}} \bigl\vert e^{-As}g \bigl(s,x(s),x(s-\tau)\bigr)-e^{-A\underline{s}}g\bigl(s,x(s),x(s-\tau)\bigr) \bigr\vert ^{2}\,ds \\& \quad \leq 4L_{2}e^{4 \vert A \vert T}T \bigl\vert e^{A(\underline{s}-s)}-I \bigr\vert ^{2}(1+2C_{1}). \end{aligned}$$

We compute the following two formulas in (3.18):

3.23 $$\begin{aligned}& E\sup_{0\leq t\leq t_{1}} \bigl\vert e^{At} \bigr\vert ^{2} \biggl\vert \int_{0}^{t}e^{-A\underline {s}}g\bigl(s,x(s),x(s-\tau) \bigr) \\& \qquad {}-e^{-A\underline {s}}g\bigl(s,z(s),z(s-\tau)\bigr)\,dB(s) \biggr\vert ^{2} \\& \quad \leq 4e^{2 \vert A \vert T}E \int_{0}^{t_{1}} \bigl\vert e^{-A\underline{s}}g \bigl(s,x(s),x(s-\tau)\bigr)-e^{-A\underline {s}}g\bigl(s,z(s),z(s-\tau)\bigr) \bigr\vert ^{2}\,ds \\& \quad \leq 16L_{1}e^{4 \vert A \vert T}TC_{2}(\xi )h+8L_{1}e^{4 \vert A \vert T} \int_{0}^{t_{1}}E\bigl( \bigl\vert x(s)-y(s) \bigr\vert ^{2} \\& \qquad {}+ \bigl\vert x(s-\tau)-y(s-\tau ) \bigr\vert ^{2}\bigr)\,ds \end{aligned}$$

and

3.24 $$\begin{aligned}& E\sup_{0\leq t\leq t_{1}} \bigl\vert e^{At} \bigr\vert ^{2} \biggl\vert \int_{0}^{t}e^{-A\underline {s}}g\bigl(s,z(s),z(s-\tau) \bigr) \\& \qquad {}-e^{-A\underline {s}}g\bigl(\underline{s},z(s),z(s-\tau)\bigr)\,dB(s) \biggr\vert ^{2} \\& \quad \leq 4e^{2 \vert A \vert T}E \int_{0}^{t} \bigl\vert e^{-A\underline{s}}g \bigl(s,z(s),z(s-\tau)\bigr)-e^{-A\underline{s}}g\bigl(\underline {s},z(s),z(s-\tau) \bigr) \bigr\vert ^{2}\,ds \\& \quad \leq 4K_{1} e^{4 \vert A \vert T}T(1+2C_{1})h. \end{aligned}$$

Substituting (3.19) - (3.24) into (3.18), we have

3.25 $$\begin{aligned}& E\sup_{0\leq t\leq t_{1}} \bigl\vert x(t)-y(t) \bigr\vert ^{2} \\& \quad \leq 6T\bigl(T+4e^{2 \vert A \vert T}\bigr)L_{2}e^{2 \vert A \vert T} \bigl\vert e^{A(\underline{s}-s)}-I \bigr\vert ^{2}(1+2C_{1}) \\& \qquad {}+12\bigl(T+4e^{2 \vert A \vert T}\bigr)L_{1}e^{2 \vert A \vert T} \int_{0}^{t_{1}}E\sup_{0\leq \nu\leq s} \bigl\vert x(\nu)-y(\nu) \bigr\vert ^{2}\,ds \\& \qquad {}+6T\bigl(T+4e^{2 \vert A \vert T}\bigr)K_{1}e^{2 \vert A \vert T}(1+2C_{1})h \\& \qquad {}+24T\bigl(T+4e^{2 \vert A \vert T}\bigr)L_{1}e^{2 \vert A \vert T}TC_{2}(\xi)h. \end{aligned}$$

By Gronwall’s inequality, since $\vert e^{A(\underline{s}-s)}-I \vert \leq \vert A \vert he^{ \vert A \vert T}$, we can show

3.26 $$\begin{aligned}& E\sup_{0\leq t\leq T} \bigl\vert x(t)-y(t) \bigr\vert ^{2} \\& \quad \leq \bigl[6e^{2 \vert A \vert T}T(T+4) \bigl(L_{2} \vert A \vert ^{2}h^{2}e^{2 \vert A \vert T}+K_{1}h\bigr) (1+2C_{1}) \\& \qquad {}+24T(T+4)L_{1}e^{2 \vert A \vert T}T(C_{2}(\xi )h \bigr]e^{12T(T+4)L_{1}e^{2 \vert A \vert T}}. \end{aligned}$$

As a result,

3.27 $$\begin{aligned} \lim_{h\rightarrow0}E\Bigl[\sup_{0\leq t\leq T} \bigl\vert y(t)-x(t) \bigr\vert ^{2}\Bigr]=0. \end{aligned}$$

The proof is completed. □

## Exponential stability in mean square

In this section, we give the exponential stability in mean square of the exact solution and the exponential Euler method to semi-linear stochastic delay differential equations (2.1). For the purpose of stability study in this paper, assume that $f(t,0,0)=g(t,0,0)=0$.

### Stability of the exact solution

In this subsection, we will show the exponential stability in mean square of the exact solution to semi-linear stochastic delay differential equations (2.1)under the global Lipschitz condition. Next we will give the main content of this subsection.

#### Theorem 4.1

*Under condition* (H1), *if* $1+2\mu[A]+4L_{1}<0$, *then the solution of equations* (2.1) *with the initial data* $\xi\in C^{b}_{\mathbf{F}_{0}}([-\tau,0];R^{n})$ *is exponentially stable in mean square*, *that is*,

4.1 $$\begin{aligned} E \bigl\vert x(t) \bigr\vert ^{2}\leq\widetilde{B}^{-1}(\tau)E \vert \xi \vert ^{2}e^{t\ln(\widetilde{B}(\tau))^{\frac{1}{2\tau}}},\quad t\geq0, \end{aligned}$$

*where* $\widetilde{B}(\tau)=e^{B_{1}\tau}-\frac{B_{2}}{B_{1}}(1-e^{B_{1}\tau })$, $B_{1}=1+2\mu[A]+2L_{1}$, $B_{2}=2L_{1}$.

By Ito’s formula and the delay term of the equation, we give the proof of Theorem 4.1. The highlight of the proof is that we give the mean square boundedness of the solution to the equation by dividing the interval into $[0,\pi],[\pi,2\pi],\ldots,[k\pi,(k+1)\pi]$. Then we give a proof of the conclusion by $t\geq0,t\geq2\pi,t\geq4\pi,\ldots,t\geq2n\pi$. In the process of dealing with the semi-linear matrix, we use the definition of the matrix norm.

#### Definition 4.1

[12]

SDDEs (2.1) are said to be exponentially stable in mean square if there is a pair of positive constants *λ* and *μ* such that for any initial data $\xi\in C^{b}_{\mathbf{F}_{0}}([-\tau,0];R^{n})$,

4.2 $$\begin{aligned} E \bigl\vert x(t) \bigr\vert ^{2}\leq\mu E \vert \xi \vert ^{2}e^{-\lambda t},\quad t\geq0. \end{aligned}$$

We refer to *λ* as the rate constant and to *μ* as the growth constant.

#### Definition 4.2

[14]

The logarithmic norm $\mu[A]$ of *A* is defined by

4.3 $$\begin{aligned} \mu[A]=\lim_{\Delta\rightarrow0^{+}}\frac{ \Vert I+\Delta A \Vert -1}{\Delta}. \end{aligned}$$

Especially, if $\Vert\cdot\Vert$ is an inner product norm, $\mu[A]$ can also be written as

4.4 $$\begin{aligned} \mu[A]=\max_{\xi\neq 0}\frac{\langle A\xi,\xi \rangle}{\Vert\xi\Vert^{2}}. \end{aligned}$$

#### Lemma 4.1

*Let* $\widetilde{B}(t)=e^{B_{1}t}-\frac{B_{2}}{B_{1}}(1-e^{B_{1}t})$. *If* $B_{1}<0$, $B_{2}>0 $ *and* $B_{1}+B_{2}<0$, *then for all* $t\geq0$, $0< \widetilde{B}(t)\leq1$ *and* $\widetilde{B}(t)$ *is decreasing*.

#### Proof

It is known from $B_{1}<0$, $B_{2}>0 $ and $B_{1}+B_{2}<0$ that for all $t\geq0$

$$\widetilde{B}(t)=\frac{B_{1}+B_{2}}{B_{1}}e^{B_{1}t}-\frac{B_{2}}{B_{1}}>0 $$

and

$$\widetilde{B}(t)=e^{B_{1}t}-1+\frac{B_{2}}{B_{1}}\bigl(e^{B_{1}t}-1 \bigr)+1=\frac {(B_{1}+B_{2})(e^{B_{1}t}-1)}{B_{1}}+1\leq1. $$

For all $t\geq0$, we compute

$$\widetilde{B}^{'}(t)=(B_{1}+B_{2})e^{B_{1}t}< 0. $$

Thus $\widetilde{B}(t)$ is decreasing. The proof is complete. □

#### Proof of Theorem 4.1

By Itô’s formula and Definition 4.2, for all $t\geq0$, we have

4.5 $$\begin{aligned} d \bigl\vert x(t) \bigr\vert ^{2} =& \bigl[\big\langle 2x(t),Ax(t)+f\bigl(t,x(t),x(t-\tau)\bigr)\big\rangle \\ &{}+ \bigl\vert g\bigl(t,x(t),x(t-\tau)\bigr) \bigr\vert ^{2} \bigr]\,dt \\ &{}+2x^{T}(t)g\bigl(t,x(t),x(t-\tau)\bigr)\,dB(t) \\ \leq&\bigl[2\big\langle x(t),Ax(t)\big\rangle +2\big\langle x(t),f\bigl(t,x(t),x(t-\tau)\bigr)\big\rangle \\ &{}+ \bigl\vert g\bigl(t,x(t),x(t-\tau)\bigr) \bigr\vert ^{2} \bigr]\,dt \\ &{}+2x^{T}(t)g\bigl(t,x(t),x(t-\tau)\bigr)\,dB(t) \\ \leq&\bigl[B_{1} \bigl\vert x(t) \bigr\vert ^{2}+B_{2} \bigl\vert x(t-\tau) \bigr\vert ^{2}\bigr]\,dt \\ &{}+2x^{T}(t)g\bigl(t,x(t),x(t-\tau)\bigr)\,dB(t), \end{aligned}$$

where $B_{1}=1+2\mu[A]+2L_{1}$, $B_{2}=2L_{1}$. Let $V(x,t)=e^{-B_{1}t} \vert x(t) \vert ^{2}$, by Itô’s formula, we obtain

4.6 $$\begin{aligned} d\bigl(e^{-B_{1}t} \bigl\vert x(t) \bigr\vert ^{2}\bigr) =&-B_{1}e^{-B_{1}t} \bigl\vert x(t) \bigr\vert ^{2}\,dt+e^{-B_{1}t}\,d \bigl\vert x(t) \bigr\vert ^{2} \\ \leq&-B_{1}e^{-B_{1}t} \bigl\vert x(t) \bigr\vert ^{2}\,dt+e^{-B_{1}t}\bigl[B_{1} \bigl\vert x(t) \bigr\vert ^{2}+B_{2} \bigl\vert x(t-\tau ) \bigr\vert ^{2}\bigr]\,dt \\ &{}+2e^{-B_{1}t}x^{T}(t)g\bigl(t,x(t),x(t-\tau)\bigr)\,dB(t) \\ \leq&e^{-B_{1}t}B_{2} \bigl\vert x(t-\tau) \bigr\vert ^{2}\,dt \\ &{}+2e^{-B_{1}t}x^{T}(t)g\bigl(t,x(t),x(t-\tau)\bigr)\,dB(t). \end{aligned}$$

Integrating (4.6) from 0 to *t* yields

4.7 $$\begin{aligned} e^{-B_{1}t} \bigl\vert x(t) \bigr\vert ^{2} \leq & \bigl\vert x(0) \bigr\vert ^{2} +B_{2} \int_{0}^{t}e^{-B_{1}s} \bigl\vert x(s-\tau) \bigr\vert ^{2}\,ds \\ &{}+2 \int_{0}^{t}e^{-B_{1}s}x^{T}(s)g \bigl(s,x(s),x(s-\tau)\bigr)\,dB(s). \end{aligned}$$

Taking expected values gives

4.8 $$\begin{aligned} e^{-B_{1}t}E \bigl\vert x(t) \bigr\vert ^{2} \leq &E \bigl\vert x(0) \bigr\vert ^{2} +B_{2} \int_{0}^{t}e^{-B_{1}s}E \bigl\vert x(s- \tau) \bigr\vert ^{2}\,ds. \end{aligned}$$

For any $t\in[0,\tau]$, we have

4.9 $$\begin{aligned} e^{-B_{1}t}E \bigl\vert x(t) \bigr\vert ^{2} \leq &E \vert \xi \vert ^{2} + E \vert \xi \vert ^{2} B_{2} \int_{0}^{t}e^{-B_{1}s}\,ds \\ \leq&\biggl[1-\frac{B_{2}}{B_{1}}\bigl(e^{-B_{1}t}-1\bigr)\biggr]E \vert \xi \vert ^{2}. \end{aligned}$$

Hence

4.10 $$\begin{aligned} E \bigl\vert x(t) \bigr\vert ^{2}\leq \biggl[e^{B_{1}t}-\frac{B_{2}}{B_{1}}\bigl(1-e^{B_{1}t}\bigr)\biggr]E \vert \xi \vert ^{2}=\widetilde{B}(t)E \vert \xi \vert ^{2}. \end{aligned}$$

For any $t\in[\tau,2\tau]$, we obtain

4.11 $$\begin{aligned} e^{-B_{1}t}E \bigl\vert x(t) \bigr\vert ^{2} \leq &e^{-B_{1}\tau}E \bigl\vert x(\tau) \bigr\vert ^{2} +B_{2} \int_{\tau}^{t}e^{-B_{1}s}E \bigl\vert x(s- \tau) \bigr\vert ^{2}\,ds \\ \leq&e^{-B_{1}\tau}\widetilde{B}(\tau)E \vert \xi \vert ^{2} +E \vert \xi \vert ^{2}B_{2} \int_{\tau}^{t}e^{-B_{1}s}\,ds \\ =&e^{-B_{1}\tau}\widetilde{B}(\tau)E \vert \xi \vert ^{2}+E \vert \xi \vert ^{2}\biggl[-\frac{B_{2}}{B_{1}}\bigl(e^{-B_{1}t}-e^{-B_{1}\tau} \bigr)\biggr]. \end{aligned}$$

Thus

4.12 $$\begin{aligned} E \bigl\vert x(t) \bigr\vert ^{2} \leq& e^{B_{1}(t-\tau)}\widetilde{B}(\tau)E \vert \xi \vert ^{2}+E \vert \xi \vert ^{2}\biggl[-\frac{B_{2}}{B_{1}}\bigl(1-e^{B_{1}(t-\tau)} \bigr)\biggr] \\ \leq&E \vert \xi \vert ^{2}\biggl[e^{B_{1}(t-\tau)}- \frac {B_{2}}{B_{1}}\bigl(1-e^{B_{1}(t-\tau)}\bigr)\biggr] \\ =&\widetilde{B}(t-\tau)E \vert \xi \vert ^{2}. \end{aligned}$$

Repeating this procedure, for all $t\in[k\tau,(k+1)\tau]$, we can show

4.13 $$\begin{aligned} E \bigl\vert x(t) \bigr\vert ^{2}\leq\widetilde {B}(t-k\tau)E \vert \xi \vert ^{2}. \end{aligned}$$

Hence, for any $t>0$, we have

4.14 $$\begin{aligned} E \bigl\vert x(t) \bigr\vert ^{2}\leq E \vert \xi \vert ^{2}. \end{aligned}$$

On the other hand, for any $t\geq0$, one can easily show that

4.15 $$ \begin{aligned}[b] e^{-B_{1}t}E \bigl\vert x(t) \bigr\vert ^{2}&\leq E \bigl\vert x(0) \bigr\vert ^{2}+B_{2} \int_{0}^{t}e^{-B_{1}s}E \bigl\vert x(s- \tau) \bigr\vert ^{2}\,ds \\ &\leq E \vert \xi \vert ^{2}+E \vert \xi \vert ^{2}B_{2} \int_{0}^{t}e^{-B_{1}s}\,ds \\ &= E \vert \xi \vert ^{2}\biggl[1-\frac{B_{2}}{B_{1}} \bigl(e^{-B_{1}t}-1\bigr)\biggr]. \end{aligned} $$

Therefore,

4.16 $$\begin{aligned} E \bigl\vert x(t) \bigr\vert ^{2}\leq E \vert \xi \vert ^{2}\biggl[e^{B_{1}t}-\frac{B_{2}}{B_{1}} \bigl(1-e^{B_{1}t}\bigr)\biggr]=\widetilde {B}(t)E \vert \xi \vert ^{2}. \end{aligned}$$

Especially, we can see

4.17 $$\begin{aligned} E \bigl\vert x(2\tau) \bigr\vert ^{2}\leq \widetilde{B}(2\tau)E \vert \xi \vert ^{2}. \end{aligned}$$

For any $t\geq2\tau$, we have

4.18 $$\begin{aligned} e^{-B_{1}t}E \bigl\vert x(t) \bigr\vert ^{2} \leq &e^{-2B_{1}\tau}E \bigl\vert x(2\tau) \bigr\vert ^{2}+B_{2} \int_{2\tau }^{t}e^{-B_{1}s}E \bigl\vert x(s- \tau) \bigr\vert ^{2}\,ds \\ \leq&e^{-2B_{1}\tau}\widetilde{B}(2\tau)E \vert \xi \vert ^{2}+B_{2} \int_{2\tau}^{t}e^{-B_{1}s}B(s-\tau)E \vert \xi \vert ^{2}\,ds \\ \leq&e^{-2B_{1}\tau}\widetilde{B}(2\tau)E \vert \xi \vert ^{2}+ \widetilde{B}(\tau)E \vert \xi \vert ^{2}B_{2} \int_{2\tau }^{t}e^{-B_{1}s}\,ds \\ \leq&e^{-2B_{1}\tau}\widetilde{B}(\tau)E \vert \xi \vert ^{2}+ \widetilde{B}(\tau)E \vert \xi \vert ^{2}\biggl[-\frac {B_{2}}{B_{1}} \bigl(e^{-B_{1}t}-e^{-2B_{1}\tau}\bigr)\biggr] \\ \leq&\widetilde{B}(\tau)E \vert \xi \vert ^{2} \biggl[e^{-2B_{1}\tau}-\frac{B_{2}}{B_{1}}\bigl(e^{-B_{1}t}-e^{-2B_{1}\tau} \bigr)\biggr]. \end{aligned}$$

Therefore,

4.19 $$\begin{aligned} E \bigl\vert x(t) \bigr\vert ^{2} \leq& \widetilde {B}(\tau)E \vert \xi \vert ^{2}\biggl[e^{B_{1}(t-2\tau)}- \frac {B_{2}}{B_{1}}\bigl(1-e^{B_{1}(t-2\tau)}\bigr)\biggr] \\ =&\widetilde{B}(\tau )\widetilde{B}(t-2\tau) E \vert \xi \vert ^{2}. \end{aligned}$$

Obviously, we can obtain

4.20 $$\begin{aligned} E \bigl\vert x(4\tau) \bigr\vert ^{2}\leq \widetilde{B}(\tau)\widetilde{B}(2\tau)E \vert \xi \vert ^{2}\leq \widetilde{B}^{2}(\tau)E \vert \xi \vert ^{2}. \end{aligned}$$

For any $t\geq4\tau$, we can see that

4.21 $$\begin{aligned} e^{-B_{1}t}E \bigl\vert x(t) \bigr\vert ^{2} \leq &e^{-4B_{1}\tau}E \bigl\vert x(4\tau) \bigr\vert ^{2}+B_{2} \int_{4\tau }^{t}e^{-B_{1}s}E \bigl\vert x(s- \tau) \bigr\vert ^{2}\,ds \\ \leq&e^{-4B_{1}\tau}\widetilde{B}(4\tau)E \vert \xi \vert ^{2}+B_{2} \int_{4\tau}^{t}e^{-B_{1}s}\widetilde{B}(\tau) \widetilde {B}(s-3\tau)E \vert \xi \vert ^{2}\,ds \\ \leq&e^{-4B_{1}\tau}\widetilde{B}^{2}(\tau)E \vert \xi \vert ^{2}+\widetilde{B}^{2}(\tau)E \vert \xi \vert ^{2}B_{2} \int _{4\tau}^{t}e^{-B_{1}s}\,ds \\ \leq&e^{-4B_{1}\tau}\widetilde{B}^{2}(\tau)E \vert \xi \vert ^{2}+\widetilde{B}^{2}(\tau)E \vert \xi \vert ^{2}\biggl[-\frac {B_{2}}{B_{1}}\bigl(e^{-B_{1}t}-e^{-4B_{1}\tau} \bigr)\biggr] \\ \leq&\widetilde{B}^{2}(\tau)E \vert \xi \vert ^{2} \biggl[e^{-4B_{1}\tau}-\frac{B_{2}}{B_{1}}\bigl(e^{-B_{1}t}-e^{-4B_{1}\tau} \bigr)\biggr]. \end{aligned}$$

Therefore,

4.22 $$ \begin{aligned}[b] E \bigl\vert x(t) \bigr\vert ^{2}&\leq \widetilde {B}^{2}(\tau)E \vert \xi \vert ^{2}\biggl[e^{B_{1}(t-4\tau)}- \frac {B_{2}}{B_{1}}\bigl(1-e^{B_{1}(t-4\tau)}\bigr)\biggr] \\ &= \widetilde{B}^{2}(\tau )\widetilde{B}(t-4\tau) E \vert \xi \vert ^{2}. \end{aligned} $$

For any $t\geq0$, there is an integer *n* such that $t\geq2n\tau$; repeating this procedure, we can show

4.23 $$\begin{aligned} E \bigl\vert x(t) \bigr\vert ^{2}\leq\widetilde {B}^{n}(\tau)\widetilde{B}(t-n\tau)E \vert \xi \vert ^{2} \leq \widetilde{B}^{n}(\tau)E \vert \xi \vert ^{2}. \end{aligned}$$

By (4.23) and Lemma 4.1, we obtain

4.24 $$\begin{aligned} E \bigl\vert x(t) \bigr\vert ^{2} \leq&\widetilde {B}^{n}(\tau)E \vert \xi \vert ^{2} \\ =& e^{2n\tau\ln(\widetilde{B}(\tau))^{\frac{1}{2\tau}}}E \vert \xi \vert ^{2} \\ =& e^{(2n\tau-t)\ln(\widetilde{B}(\tau ))^{\frac{1}{2\tau}}}E \vert \xi \vert ^{2}e^{t\ln(\widetilde {B}(\tau))^{\frac{1}{2\tau}}} \\ \leq&e^{-2\tau\ln(\widetilde {B}(\tau))^{\frac{1}{2\tau}}}E \vert \xi \vert ^{2}e^{t\ln (\widetilde{B}(\tau))^{\frac{1}{2\tau}}} \\ =&\widetilde{B}^{-1}(\tau)E \vert \xi \vert ^{2}e^{t\ln(\widetilde{B}(\tau))^{\frac{1}{2\tau}}}, \end{aligned}$$

which proves the theorem. □

### Stability of the exponential Euler method

In this subsection, under the same conditions as those in Theorem 4.1, we will obtain the exponential stability in mean square of the exponential Euler method (2.4) to SLSDDEs (2.1) in Theorem 4.2. It is shown that the stability region of the numerical solution to the equation is the same as that of the analytical solution, which means that our method is effective.

#### Definition 4.3

[12]

Given a step size $h=\tau/m$ for some positive integer *m*, the discrete exponential Euler method is said to be exponentially stable in mean square on SDDEs (2.1) if there is a pair of positive constants *λ̄* and *μ̄* such that for any initial data $\xi\in C^{b}_{\mathbf{F}_{0}}([-\tau,0];R^{n})$,

4.25 $$\begin{aligned} E \vert y_{n} \vert ^{2}\leq\bar{\mu} E \vert \xi \vert ^{2}e^{-\bar{\lambda} nh},\quad n\geq0. \end{aligned}$$

#### Lemma 4.2

[14]

*Let* $\mu[A]$ *be the smallest possible one*-*sided Lipschitz constant of the matrix* *A* *for a given inner product*. *Then* $\mu[A]$ *is the smallest element of the set*

4.26 $$\begin{aligned} M=\bigl\{ \theta: \bigl\Vert \exp(At) \bigr\Vert \leq\exp (\theta t),t\geq0 \bigr\} . \end{aligned}$$

#### Theorem 4.2

*Under condition* (H1), *if* $1+2\mu [A]+4L_{1}<0$, *then for all* $h>0$ *the numerical method to equations* (2.1) *is exponentially stable in mean square*, *that is*,

4.27 $$\begin{aligned} E \vert y_{n} \vert ^{2}\leq(A_{1}+A_{2})^{-1}E \vert y_{0} \vert ^{2}e^{nh\ln(A_{1}+A_{2})^{\frac{1}{2\tau}}}, \end{aligned}$$

*where* $A_{1}=e^{2\mu[A]h}(1+L_{1}h^{2}+2L_{1}h+h)$, $A_{2}=e^{2\mu[A]h}(L_{1}h^{2}+2L_{1}h)$.

#### Proof

Squaring and taking the conditional expectation on both sides of (2.3), noting that $\Delta B_{n}$ is independent of $\mathbf{F}_{nh}$, $E(\Delta B_{n}\vert\mathbf{F}_{nh})=E(\Delta B_{n})=0$ and $E((\Delta B_{n})^{2}\vert\mathbf{F}_{nh})=E(\Delta B_{n})^{2}=h$, we have

4.28 $$\begin{aligned} E\bigl( \vert y_{n+1} \vert ^{2}\vert \mathbf{F}_{nh}\bigr) =&e^{2\mu [A]h}E \vert y_{n} \vert ^{2} +e^{2\mu[A]h}E\bigl( \bigl\vert f(t_{n},y_{n},y_{n-m}) \bigr\vert ^{2}\vert\mathbf {F}_{nh}\bigr)h^{2} \\ &{}+e^{2\mu[A]h}E\bigl( \bigl\vert g(t_{n},y_{n},y_{n-m}) \bigr\vert ^{2}\vert\mathbf{F}_{nh}\bigr)h \\ &{}+2e^{2\mu[A]h}E\bigl(\bigl\langle y_{n},f(t_{n},y_{n},y_{n-m})\bigr\rangle \vert\mathbf{F}_{nh}\bigr)h. \end{aligned}$$

Taking expectations on both sides, we obtain that

4.29 $$\begin{aligned} E \vert y_{n+1} \vert ^{2} =&e^{2\mu[A]h}E \vert y_{n} \vert ^{2}+e^{2\mu[A]h}E \bigl\vert f(t_{n},y_{n},y_{n-m}) \bigr\vert ^{2}h^{2} \\ &{}+e^{2\mu[A]h}E \bigl\vert g(t_{n},y_{n},y_{n-m}) \bigr\vert ^{2}h \\ &{}+2e^{2\mu[A]h}E\bigl\langle y_{n},f(t_{n},y_{n},y_{n-m})\bigr\rangle h. \end{aligned}$$

By (H1) and the inequality $2ab\leq a^{2}+b^{2}$, we have

4.30 $$\begin{aligned} 2E\bigl\langle y_{n},f(t_{n},y_{n},y_{n-m})\bigr\rangle \leq& E \vert y_{n} \vert ^{2}+E \bigl\vert f(t_{n},y_{n},y_{n-m}) \bigr\vert ^{2} \\ \leq& (1+L_{1})E \vert y_{n} \vert ^{2}+L_{1}E \vert y_{n-m}) \vert ^{2}. \end{aligned}$$

Substituting (4.30) into (4.29), by (H1), we have

4.31 $$\begin{aligned} E \vert y_{n+1} \vert ^{2} \leq& e^{2\mu [A]h}\bigl[\bigl(1+L_{1}h^{2}+2L_{1}h+h \bigr)E \vert y_{n} \vert ^{2}+\bigl(L_{1}h^{2}+2L_{1}h \bigr)E \vert y_{n-m} \vert ^{2}\bigr] \\ =&A_{1}E \vert y_{n} \vert ^{2}+A_{2}E \vert y_{n-m} \vert ^{2}, \end{aligned}$$

where $A_{1}=e^{2\mu[A]h}(1+L_{1}h^{2}+2L_{1}h+h)$, $A_{2}=e^{2\mu[A]h}(L_{1}h^{2}+2L_{1}h)$. In view of $1+2\mu[A]+4L_{1}<0$, we have $\mu[A]<0$ and $-\mu[A]>\max\{1,L_{1}\}$. Consequently, $L_{1}-\mu^{2}[A]<0$. Hence

4.32 $$\begin{aligned} 2\bigl(L_{1}-\mu^{2}[A]\bigr)h+1+2 \mu[A]+4L_{1}< 0 \end{aligned}$$

for all $h>0$, which implies

4.33 $$\begin{aligned} 1+h+4L_{1}h+2L_{1}h^{2}< 1-2\mu[A]h+ \frac{(-2\mu [A]h)^{2}}{2!}< e^{-2\mu[A]h}. \end{aligned}$$

That is,

4.34 $$\begin{aligned} A_{1}+A_{2}=e^{2\mu[A]h} \bigl(1+h+4L_{1}h+2L_{1}h^{2}\bigr)< 1 \end{aligned}$$

for all $h>0$. From (4.31), we have

4.35 $$\begin{aligned} E \vert y_{n} \vert ^{2} \leq(A_{1}+A_{2})^{[\frac{n}{m+1}]+1}E \vert y_{0} \vert ^{2}. \end{aligned}$$

So we obtain

4.36 $$ \begin{aligned}[b] E \vert y_{n} \vert ^{2}&\leq (A_{1}+A_{2})^{[\frac {n}{m+1}]+1}E \vert y_{0} \vert ^{2} \\ &=e^{([\frac{n}{m+1}]+1)\ln(A_{1}+A_{2})}E \vert y_{0} \vert ^{2} \\ &\leq e^{[\frac{n}{m+1}](m+1)h\ln(A_{1}+A_{2})^{\frac {1}{(m+1)h}}}E \vert y_{0} \vert ^{2} \\ &\leq e^{-\{\frac{n}{m+1}\}(m+1)h\ln(A_{1}+A_{2})^{\frac {1}{(m+1)h}}}E \vert y_{0} \vert ^{2}e^{nh\ln(A_{1}+A_{2})^{\frac{1}{(m+1)h}}} \\ &\leq e^{-(m+1)h\ln(A_{1}+A_{2})^{\frac{1}{(m+1)h}}}E \vert y_{0} \vert ^{2}e^{nh\ln(A_{1}+A_{2})^{\frac{1}{(m+1)h}}} \\ &=(A_{1}+A_{2})^{-1}E \vert y_{0} \vert ^{2}e^{nh\ln (A_{1}+A_{2})^{\frac{1}{(m+1)h}}} \\ &=(A_{1}+A_{2})^{-1}E \vert y_{0} \vert ^{2}e^{nh\ln (A_{1}+A_{2})^{\frac{1}{2\tau}}}. \end{aligned} $$

Thus, for all $n=1,2\ldots$ ,

4.37 $$\begin{aligned} E \vert y_{n} \vert ^{2} \leq(A_{1}+A_{2})^{-1}E \vert y_{0} \vert ^{2}e^{nh\ln(A_{1}+A_{2})^{\frac{1}{2\tau}}}. \end{aligned}$$

The proof is completed. □

## Numerical experiments

In this section, we give several numerical experiments in order to demonstrate the results about the strong convergence and the exponential stability in mean square of the numerical solution for equations (2.1). We consider the test equation

5.1 $$\begin{aligned} dx(t)=\bigl[a_{1}x(t)+a_{2}x(t-\tau)\bigr]\,dt+ \bigl[b_{1}x(t)+b_{2}x(t-\tau)\bigr]\,d B(t)\quad \forall t \geq0. \end{aligned}$$

### Example 5.1

When $a_{1}=-4$, $a_{2}=1.5$, $b_{1}=1$, $b_{2}=0.05$, $\xi=1+t$, $\tau=1$. In Table 1, the convergence of the exponential Euler method to Example 5.1 is described. Here we focus on the error at the endpoint $T=2,4$, and the error is given as $E \vert y_{n}(\omega)-x(T,\omega) \vert ^{2}$, where $y_{n}(\omega)$ denotes the value of (2.3) at the endpoint. The expectation is estimated by averaging random sample paths ($\omega_{i}$, $1\leq i\leq1\text{,}000$) over the interval $[0,10]$, that is,

$$e(h)=\frac{1}{1\text{,}000}\sum_{i=1}^{11\text{,}000} \bigl\vert y_{n}(\omega_{i})-x(T,\omega_{i}) \bigr\vert ^{2}. $$

In Table 1, we can see that the exponential Euler method to Example 5.1 is convergent, suggesting that (2.3) is valid.

**Table 1:** **The global error of numerical solutions for the exponential Euler method**

**Step size**	$\boldsymbol{\epsilon_{2}}$	$\boldsymbol{\epsilon_{4}}$
$h=\frac{1}{2}$	0.11758788103726	0.05128510485760
$h=\frac{1}{4}$	0.01076456781468	0.00178190502421
$h=\frac{1}{8}$	4.226428624973588e − 004	2.606318250482847e − 004
$h=\frac{1}{16}$	1.080022443102593e − 004	6.629709170569013e − 005
$h=\frac{1}{32}$	1.325175503903862e − 005	1.152618733195335e − 005
$h=\frac{1}{64}$	3.097379961005242e − 007	2.047860964726653e − 006
$h=\frac{1}{128}$	8.055605114301942e − 009	5.371039941796389e − 007

### Example 5.2

**Figure 1:**
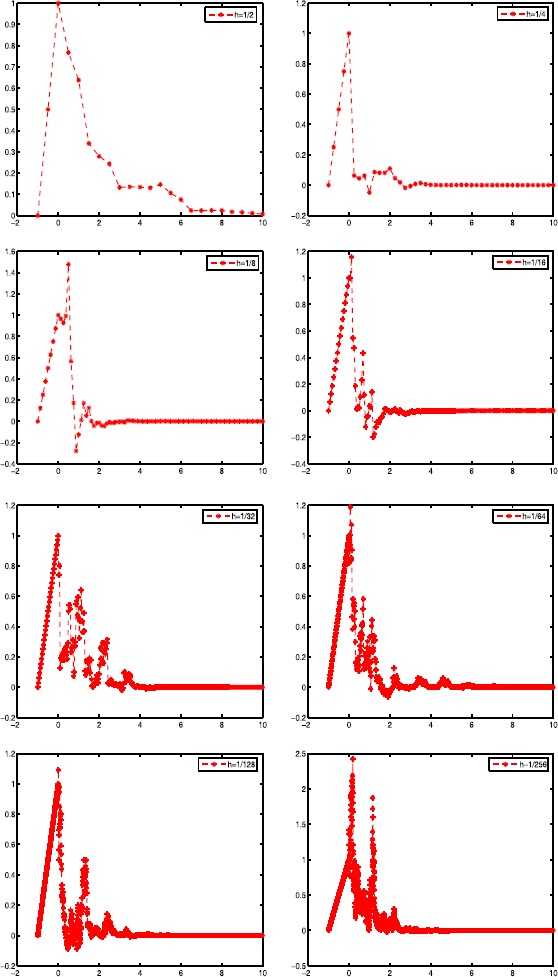
**The numerical solutions with** $\pmb{h=1/2,1/4,1/8,1/16,1/32,1/64,1/128,1/256}$ **for EEM.**

When $a_{1}=-5$, $a_{2}=1$, $b_{1}=2$, $b_{2}=0.5$, $\xi=1+t$, $\tau=1$. We can show the stability of the exponential Euler method to (2.3). In Figure 1, all the curves decay toward to zero when $h=1/2$, $h=1/4$, $h=1/8$, $h=1/16$, $h=1/32$, $h=1/64$, $h=1/128$, $h=1/256$. So we can consider that our experiments are consistent with our proved results in Section 4.

## Conclusions

In this paper, we study convergence and exponential stability in mean square of the numerical solution for the exponential Euler method to semi-linear stochastic delay differential equations under the global Lipschitz condition and the linear growth condition. Firstly, Theorem 3.1 gives the exponential Euler approximation solution converging to the analytic solution with the strong order $\frac{1}{2}$ to SLSDDEs. Secondly, we give the exponential stability in mean square of the exact solution to SLSDDEs by using the definition of logarithmic norm. Then we propose an explicit method to show that the exponential Euler method to SLSDDEs is proved to share the same stability for any step size. Finally, a numerical example is given to verify the method, the conclusion is correct. In Table 1, the convergence of the exponential Euler method to Example 5.1 is described. Here we focus on the error at the endpoint $T=2,4$. In Figure 1, all the curves decay toward zero when $h=1/2$, $h=1/4$, $h=1/8$, $h=1/16$, $h=1/32$, $h=1/64$, $h=1/128$, $h=1/256$, and there is the same conclusion for any step size. So we can consider that our experiments are consistent with our proved results in Section 4.

## References

[1] Friedman A (1975). Stochastic Differential Equations and Applications, Vol. 1 and 2.

[2] Higham DJ, Mao X, Yuan C (2007). Almost sure and moment exponential stability in the numerical simulation of stochastic differential equations. SIAM J. Numer. Anal..

[3] Mao X (1997). Stochastic Differential Equations and Applications.

[4] Fuke W, Xuerong M (2014). Convergence and stability of the semi-tamed Euler scheme for stochastic differential equations with non-Lipschitz continuous coefficients. Appl. Math. Comput..

[5] Mao X (2015). The truncated Euler Maruyama method for stochastic differential equations. J. Comput. Appl. Math..

[6] Mao X (2016). Convergence rates of the truncated Euler Maruyama method for stochastic differential equations. J. Comput. Appl. Math..

[7] Mao X (2015). Almost sure exponential stability in the numerical simulation of stochastic differential equations. SIAM J. Numer. Anal..

[8] Cao WR, Liu MZ, Fan ZC (2004). MS-stability of the Euler-Maruyama method for stochastic differential delay equations. Appl. Math. Comput..

[9] Fan ZC, Liu MZ (2005). The Pth moment exponential stability for the stochastic delay differential equation. J. Nat. Sci. Heilongjiang Univ..

[10] Mao X (2011). Numerical solutions of stochastic differential delay equations under the generalized Khasminskii-type conditions. Appl. Math. Comput..

[11] Wu* K, Ding X (2014). Convergence and stability of Euler method for impulsive stochastic delay differential equations. Appl. Math. Comput..

[12] Mao X (2007). Exponential stability of equidistant Euler-Maruyama approximations of stochastic differential delay equations. J. Comput. Appl. Math..

[13] Kunze M, Neerven J (2011). Approximating the coefficients in semilinear stochastic partial differential equations. J. Evol. Equ..

[14] Dekker K, Verwer JG (1983). Stability of Runge-Kutta Methods for Stiff Nonlinear Differential Equations.

